# Impaired Systemic Tetrahydrobiopterin Bioavailability and Increased Dihydrobiopterin in Adult Falciparum Malaria: Association with Disease Severity, Impaired Microvascular Function and Increased Endothelial Activation

**DOI:** 10.1371/journal.ppat.1004667

**Published:** 2015-03-12

**Authors:** Tsin W. Yeo, Daniel A. Lampah, Enny Kenangalem, Emiliana Tjitra, Ric N. Price, J. Brice Weinberg, Keith Hyland, Donald L. Granger, Nicholas M. Anstey

**Affiliations:** 1 Global and Tropical Health Division, Menzies School of Health Research and Charles Darwin University, Darwin, Northern Territory, Australia; 2 Lee Kong Chian School of Medicine, Nanyang Technological University, Singapore; 3 Institute of Infectious Disease and Epidemiology, Tan Tock Seng Hospital, Singapore; 4 Menzies School of Health Research-National Institute of Health Research and Development Research Program, and District Ministry of Health, Timika, Papua, Indonesia; 5 National Institute of Health Research and Development, Jakarta, Indonesia; 6 Centre for Tropical Medicine, Nuffield Department of Clinical Medicine, University of Oxford, Oxford, United Kingdom; 7 Duke University and VA Medical Centers, Durham, North Carolina, United States of America; 8 Medical Neurogenetics LLC, Atlanta, Georgia, United States of America; 9 Division of Infectious Diseases, University of Utah and Veterans Affairs Medical Center, Salt Lake City, Utah, United States of America; 10 Division of Medicine, Royal Darwin Hospital, Darwin, Northern Territory, Australia; Albert Einstein College of Medicine, UNITED STATES

## Abstract

Tetrahydrobiopterin (BH_4_) is a co-factor required for catalytic activity of nitric oxide synthase (NOS) and amino acid-monooxygenases, including phenylalanine hydroxylase. BH_4_ is unstable: during oxidative stress it is non-enzymatically oxidized to dihydrobiopterin (BH_2_), which inhibits NOS. Depending on BH_4_ availability, NOS oscillates between NO synthase and NADPH oxidase: as the BH_4_/BH_2_ ratio decreases, NO production falls and is replaced by superoxide. In African children and Asian adults with severe malaria, NO bioavailability decreases and plasma phenylalanine increases, together suggesting possible BH_4_ deficiency. The primary three biopterin metabolites (BH_4_, BH_2_ and B_0_ [biopterin]) and their association with disease severity have not been assessed in falciparum malaria. We measured pterin metabolites in urine of adults with severe falciparum malaria (SM; n=12), moderately-severe malaria (MSM, n=17), severe sepsis (SS; n=5) and healthy subjects (HC; n=20) as controls. In SM, urinary BH_4_ was decreased (median 0.16 ¼mol/mmol creatinine) compared to MSM (median 0.27), SS (median 0.54), and HC (median 0.34)]; p<0.001. Conversely, BH_2_ was increased in SM (median 0.91 ¼mol/mmol creatinine), compared to MSM (median 0.67), SS (median 0.39), and HC (median 0.52); p<0.001, suggesting increased oxidative stress and insufficient recycling of BH2 back to BH4 in severe malaria. Overall, the median BH_4_/BH_2_ ratio was lowest in SM [0.18 (IQR: 0.04-0.32)] compared to MSM (0.45, IQR 0.27-61), SS (1.03; IQR 0.54-2.38) and controls (0.66; IQR 0.43-1.07); p<0.001. In malaria, a lower BH_4_/BH_2_ ratio correlated with decreased microvascular reactivity (r=0.41; p=0.03) and increased ICAM-1 (r=-0.52; p=0.005). Decreased BH4 and increased BH_2_ in severe malaria (but not in severe sepsis) uncouples NOS, leading to impaired NO bioavailability and potentially increased oxidative stress. Adjunctive therapy to regenerate BH4 may have a role in improving NO bioavailability and microvascular perfusion in severe falciparum malaria.

## Author Summary

Vascular nitric oxide (NO) bioavailability is decreased in severe falciparum malaria and associated with microvascular dysfunction and increased endothelial activation. Nitric oxide synthase (NOS) requires tetrahydrobiopterin (BH_4_) as a co-factor to convert L-arginine to NO, but when BH_4_ is low, NOS is “uncoupled” and produces superoxide instead of NO. In conditions of increased oxidative stress, BH_4_ is converted to dihydrobiopterin (BH_2)_ and biopterin (B_0_): the resulting BH_2_ competes with remaining BH_4_ as a competitive inhibitor of NOS, further decreasing NO production. We measured BH_4_ and BH_2_ in the urine of adults with severe and uncomplicated falciparum malaria and compared results to those of controls or those with sepsis. There was a significant decrease in urinary BH_4_ and increase in BH_2_ in severe malaria compared to uncomplicated malaria, sepsis, and controls, suggesting increased oxidative stress and insufficient recycling of BH_2_ back to BH_4_. The BH_4_/BH_2_ ratio was associated with increased risk of severe disease, endothelial activation and microvascular dysfunction, likely through impaired NOS function. This additional mechanism of decreased NO in severe malaria suggests that trials evaluating use of adjunctive L-arginine to increase NO in severe malaria may require concurrent therapy to regenerate BH_4_.

## Introduction

Malaria remains the most important parasitic infection in humans, causing an estimated 207 million cases and 627,000 deaths in 2010 [1,2]. Mortality from severe *Plasmodium falciparum* malaria has decreased with use of intravenous artesunate, but case fatality rates remain at 8% and 15% for African children and Asian adults [3,4]. Improved understanding of the pathogenesis of severe falciparum malaria may allow identification of new targets for adjunctive therapy.

Decreased nitric oxide (NO) bioavailability is associated with increased disease severity in African children as well as Asian adults and children with falciparum malaria [5–7], but the full reasons for this observation are not known. Mechanisms identified to date include low levels of L-arginine [the substrate for NO synthase (NOS)] [6,8], impaired mononuclear cell NOS2 expression [5], inhibition of NOS by ADMA [9,10], and quenching of NO by increased plasma cell-free hemoglobin [11]. In Asian adults with moderately severe falciparum malaria (MSM), L-arginine infusion increased endothelial NO and pulmonary NO bioavailability [6]. However, a pilot trial of low-dose L-arginine infusion in adult severe falciparum malaria (SM) did not result in improvement in endothelial NO bioavailability or lactate clearance [12]. While greater L-arginine clearance in severe malaria suggest that higher doses may be more effective [12], additional mechanisms beyond L-arginine deficiency are likely to be involved.

Tetrahydrobiopterin (BH_4_) is an obligate co-factor for NO synthesis by NOS [13,14]. BH_4_ stabilizes the homodimeric NOS enzyme and participates in L-arginine oxidation and heme-iron reduction for NO production. NOS lacking BH_4_ remains catalytically active, transferring electrons from NADPH to dioxygen to produce superoxide [14,15]. Conversion of NOS catalysis from NO synthesis to superoxide production under conditions of low or absent BH_4_ is termed “uncoupling,” meaning that NADPH consumption and oxygen activation are no longer “coupled” to BH_4_-dependent L-arginine oxygenation [14,15]. In an oxidizing environment, NOS uncoupling may be related to the instability of BH_4_ because this reduced pterin spontaneously oxidizes to quininoid-BH_2_, which rapidly rearranges to the stable metabolite 7,8-dihydrobiopterin (BH_2_) that is inactive as a cofactor for NO synthesis. BH_2_ can be reduced back to BH_4_ via a tetrahydrofolate-dependent salvage pathway [16]. However if BH_2_ levels rise at the expense of BH_4_ oxidation, BH_2_ competes with BH_4_ at the NOS active site leading to NOS uncoupling and superoxide production. In cardiovascular disease, an increased BH_4_/BH_2_ ratio (as opposed to the BH_4_ concentration alone) has been found to be the best correlate for endothelial cell-dependent NO synthesis [14,15].

BH_4_ is also a co-factor for the enzyme phenylalanine hydroxylase that converts phenylalanine to tyrosine in the liver. We have found in both African children with cerebral malaria (CM) and Asian adults with SM that plasma phenylalanine levels are markedly increased [17]. We hypothesized that in SM the systemic level of BH_4_, relative to the oxidized biopterin species (BH_2_ + B_0_), would be decreased. This could explain depression in both phenylalanine hydroxylase activity and NOS functionality in severe malaria. Biopterin oxidation states in plasma and urine (which reflect systemic levels) have not been measured in malaria. Therefore we undertook measurements of urinary BH_4_, BH_2_ and B_0_ in Indonesian adults with SM and MSM and compared these to levels in healthy controls and a group presenting with severe sepsis. We hypothesized that (a) BH_4_ levels and BH_4_/BH_2_ ratios would be decreased, and BH_2_ increased in proportion to malaria disease severity, and (b) decreased BH_4_/BH_2_ ratios would be associated with increased endothelial activation and impaired NO-dependent microvascular reactivity.

## Results

### Patients

The clinical features of these subjects have previously been described [18]. We measured urinary pterin metabolites in their various oxidized states [(including biopterin (B_0_), 7, 8 dihydrobiopterin (BH_2_), tetrahydrobiopterin (BH_4_), dihydroneopterin (NH_2_) and neopterin (N_0_)] levels in 12 adults with severe malaria (SM) and 17 with moderately severe malaria (MSM), with 20 healthy adults (HCs) and 5 with severe sepsis (SS) as controls. In SM patients, 5 had single organ dysfunction (4 with cerebral malaria and 1 with acute renal failure), while the remaining 7 had two or more severity criteria. All SM and MSM patients received intravenous artesunate. In SS patients, two had pneumonia and gastroenteritis, and one each had pneumonia, gastroenteritis, and meningitis. There were 4 deaths in the SM group, and none in the MSM and SS patients. The baseline demographic details, clinical features, hematological and biochemical results of the patients are summarized in Table 1.

**Table 1:** Baseline demographics, clinical and laboratory measurements.

	Healthy Controls	Moderately-severe malaria	Severe Malaria	Severe Sepsis
Number	20	17	12	5
Age; years, (median, IQR)	29 (21–35)	28 (23–32)	30 (21–37)	25 (24–26)
Males; (number, percentage)	18 (90%)	11 (65%)	9 (75%)	1 (20%)
Days of fever before presentation (median, IQR)	NA	2 (2–5)	3 (2–5)	2 (2–5)
Systolic Blood Pressure; median (IQR), (mmHg)*	117 (113–126)	106 (100–115)	110 (101–132)	99 (94–101)
Diastolic Blood Pressure; median (IQR), (mmHg)*	70 (65–75)	59 (57–64)	64 (61–73)	64 (60–70)
Pulse Rate; median (IQR), (beats/min)*	65 (60–73)	80 (75–88)	90 (80–97)	104 (103–104)
Pulse Oxygen Saturation; mean (range), (% saturation)	99 (96–100)	98 (94–100)	96 (75–100)	99 (96–100)
Temperature; mean (range) (°Celsius)*	36.1 (35–37)	37.1 (35.9–38.9)	37.0 (35.8–39.6)	37.2 (36.7–38.0)
Leukocyte count; median (IQR), x10^3^/μL*	6.1 (4.5–6.8)	4.2 (3.7–5.8)	9.8 (7.4–12.0)	18.1 (15.4–22.1)
Hemoglobin; mean (range), (g/dl)*	12.8 (11.2–15.5)	11.5 (9.4–13.3)	12.0 (11.2–13.9)	9.2 (8.8–11.9)
Platelet; median (IQR), x10^9^/L *	153 (110–200)	53 (48–80)	26 (20–98)	96 (76–201)
Creatinine; median (IQR), (μmol/L)* (normal range: 50 to 110 μmol)	NA	87 (74–106)	182.5 (109–330)	80 (57–230)
Lactate; median ((IQR), (mmol/L)* (normal range: 0 to 2mmol)	NA	1.08 (0.9–1.8)	3.11 (1.9–5.1)	1.31 (1.2–1.5)
Parasite Density; Geometric Mean (95%CI), (parasite/μL)*	NA	14512 (8103–25989)	29269 (6453–132740)	NA
Histidine Rich Protein 2; Median (IQR), (log_e_ ng/ml)	NA	2.76 (1.94–3.22)	4.26 (3.79–6.86)	NA

*p<0.05 (for ANOVA, Kruskal-Wallis or χ^2^ test comparing severe malaria, moderately-severe malaria, severe sepsis and healthy controls)

### BH_4_, BH_2_, BH_4_/BH_2_ ratio, B_0_, NH_2_, N_0_ and clinical disease

**Fig 1:**
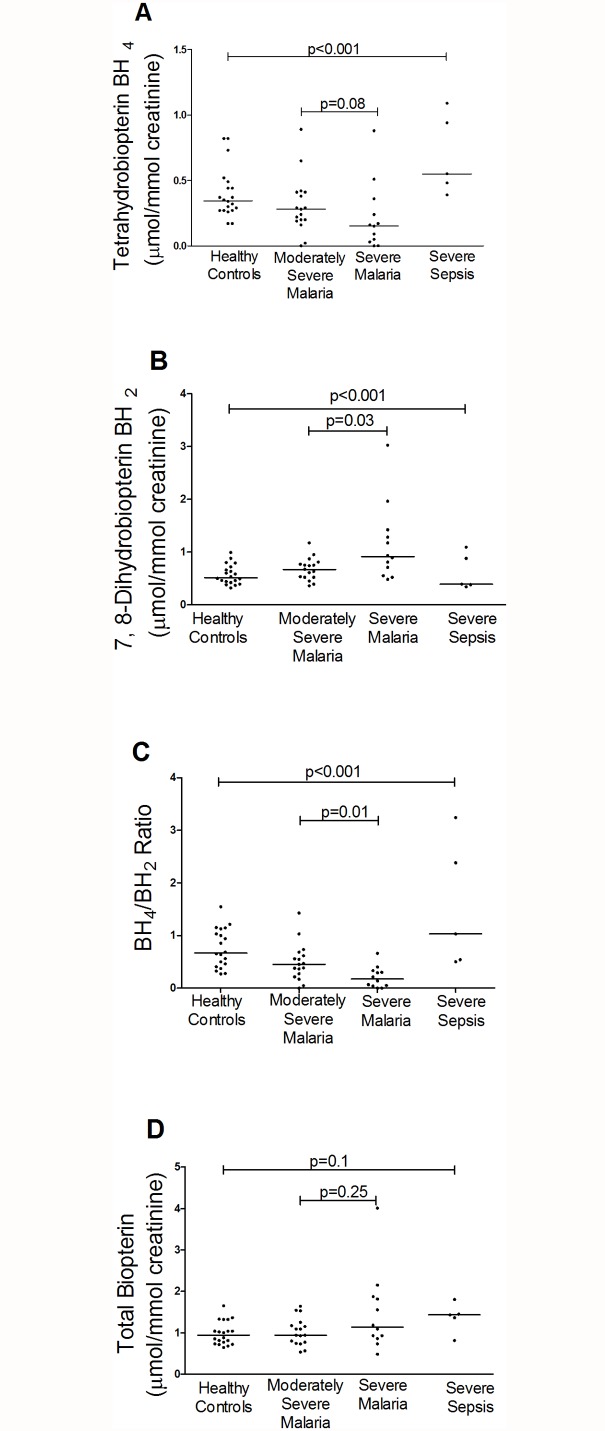
Urinary BH4, BH2 concentrations, BH4/BH2 ratios and Biopterin concentration in each group on enrollment (Kruskal-Wallis: p<0.001). Horizontal bars represent pairwise comparisons between disease groups. A. Urine BH4 (μmol/mmol creatinine) concentrations in each group on enrollment. Horizontal lines indicate median values for each group. B. Urine BH2 (μmol/mmol creatinine) concentrations in each group on enrollment. Horizontal lines indicate median values for each group. C. BH4/BH2 ratio in each group on enrollment. Horizontal lines indicate median values for each group. D. Urine Biopterin (μmol/mmol creatinine) concentrations in each group on enrollment. Horizontal lines indicate median values for each group. Horizontal bars represent pairwise comparisons between disease groups.

BH_4_ was decreased in patients with SM (median 0.16 μmol/mmol creatinine; IQR 0.04–0.30) compared to those with MSM (0.27, IQR 0.19–0.41), SS (0.54; IQR 0.48–0.94), and controls (0.34; IQR 0.27–0.46); Kruskal-Wallis p<0.001 (Table 2, Fig. 1A). In contrast, BH_2_ was increased in SM (median 0.91 μmol/mmol creatinine; IQR 0.62–1.35) compared to MSM (0.67; IQR 0.52–0.76), SS (0.39; IQR 0.38–0.88) and HCs (0.52: IQR 0.43–0.69); Kruskal-Wallis p<0.001 (Table 2, Fig. 1B). The BH_4_/BH_2_ ratio was also decreased in patients with SM (median 0.17; IQR 0.04–0.32) compared to those with MSM (0.45, IQR 0.27–61), SS (1.03; IQR 0.54–2.38) and controls (0.66; IQR 0.43–1.07); Kruskal-Wallis p<0.001 (Table 2, Fig. 1C). Conversely NH_2_ and N_0_ levels were increased in SM compared to MSM, SS, and HCs (p<0.001) (Table 2), but there were no significant differences in the total biopterin (BH_4_+BH_2_+B_0_) levels among groups (p = 0.1) (Table 2, Fig. 1D). The ratio of reduced:oxidized neopterin (NH_2_:N_0_) was 4.4 in healthy controls compared to 2.0 in severe malaria (p = 0.002, Table 2). In the 29 patients with malaria, an increased BH_4_/BH_2_ ratio was associated with severe disease (p = 0.03), however no significant associations were found for BH_4_, BH_2_, B_0,_ total biopterin, NH_2,_ N_0_ and total neopterin. The risk of death in malaria was not associated with levels of any of the pterin metabolites. There was no association between serum creatinine and urinary BH_4_, BH_2_, N_0_ and NH_2_ in patients with malaria and in the groups with severe or uncomplicated disease. On controlling for blood creatinine, there was still a significant difference in urinary BH_2_ (p = 0.011) and BH_4_/BH_2_ (p = 0.04) levels but not BH_4_ between the groups.

**Table 2:** Microvascular reactivity, endothelial function and biopterin metabolite values among patient groups.

	Healthy Controls	P value†	Moderately-severe malaria	P value††	Severe Sepsis	P value†††	Severe Malaria
Number	20		17		5		12
Recovery StO_2_%second* (StO_2recov_) Median (IQR) *	3.5 (3.0–3.7)	0.04	3.2 (2.75–3.85)	0.1	3.1 (2.6–4.0)	0.05	2.8 (1.8–3.4)
Reactive Hyperemia Peripheral Arterial Tonometry*	1.76 (1.45–2.15)	0.06	1.77 (1.6–2.2)	0.04	1.56 (1.36–1.68)	0.2	1.38 (1.27–1.87)
Tetrahydrobiopterin (BH_4_) *	0.34 (0.27–0.46)	0.006	0.27 (0.19–0.41)	0.08	0.54 (0.48–0.94)	0.008	0.16 (0.04–0.30)
7, 8 dihydrobiopterin (BH_2_) *	0.52 (0.43–0.69)	0.002	0.67 (0.52–0.76)	0.03	0.39 (0.38–0.88)	0.05	0.91 (0.62–1.35)
B_0_ Biopterin*	0.01 (0–0.02)	0.02	0.02 (0.01–0.03)	0.08	0.03 (0.02–0.07)	0.08	0.06 (0.01–0.19)
Urine Total Biopterins (BH_4_ + BH_2_ + B_0_)	0.94 (0.75–1.18)	0.09	0.94 (0.76–1.17)	0.2	1.42 (1.35–1.45)	0.6	1.14 (0.89–1.84)
BH_4_/BH_2_ ratio*	0.67 (0.43–1.08)	0.001	0.45 (0.27–0.62)	0.01	1.03 (0.54–2.38)	0.003	0.18 (0.04–0.32)
Dihydroneopterin (NH_2_) *	1.73 (0.95–3.20)	0.001	6.9 (4.90–8.00)	0.6	3.02 (2.57–4.80)	0.1	6.2 (4.35–9.78)
Neopterin (N_0_) *	0.45 (0.21–0.72)	<0.0001	1.86 (1.29–2.84)	0.03	0.85 (0.77–1.47)	0.02	4.16 (2.04–5.11)
Total Neopterin (NH_2_ + N_0_) *	2.19 (1.16–3.90)	<0.0001	8.65 (6.40–10.67)	0.2	3.87 (3.3–6.24)	0.03	10.78 (6.55–13.60)
NH2/N_0_ Ratio	4.44 (2.52–8)	0.002	3.57 (2.32–5.38)	0.03	3.23 (2.9–3.58)	0.17	1.98 (0.54–5.48)
Phenylalanine (μmol/L) *	54 (51–58)	0.03	101 (84–110)	0.09	114 (112–332)	0.2	176 (85–250)

Median and IQR unless otherwise stated. All pterin measurements are μmol/mmol creatinine

*p<0.05 (for ANOVA, Kruskal-Wallis or χ^2^ test comparing severe malaria, moderately-severe malaria, severe sepsis and healthy controls)

†Pairwise comparisons between each group and severe malaria using Wilcoxon-Mann-Whitney test: † control vs severe malaria;

†† moderately severe malaria vs severe malaria;

††† severe sepsis vs severe malaria.

### BH_4_, BH_2_, BH_4_/BH_2_ ratio, and biomarkers of severity

Peripheral parasitemia was correlated with increasing BH_2_ (r = 0.46, p = 0.01) and N_0_ (r = 0.50, p = 0.006) levels, and parasite biomass (estimated using plasma HRP2) was positively correlated with BH_2_ (r = 0.44, p = 0.02), and inversely with the BH_4_/BH_2_ ratio (r = -0.41, p = 0.03) in all malaria patients but not after controlling for malarial disease severity. Increasing venous lactate was associated with higher BH_2_ levels (r = 0.48, p = 0.008) and a lower BH_4_/BH_2_ ratio (r = -0.43, p = 0.01) in all malaria patients but not after controlling for severity of disease.

**BH_4_, BH_2_, BH_4_/BH_2_ ratio, microvascular reactivity, and endothelial activation**. Similar to our previous published results [18], microvascular reactivity and endothelial function were reduced in SM compared to MSM and HCs (Table 2). In all malaria patients, higher microvascular reactivity was associated with an increased BH_4_/BH_2_ ratio (r = 0.41, p = 0.03) and lower BH_2_ levels (r = -0.42, p = 0.024), with no association found for the other biopterin metabolites. The associations with the BH_4_/BH_2_ ratio and BH_2_ remained significant after controlling for disease severity (partial correlation coefficient = 0.34, p = 0.04 and partial correlation coefficient = -0.38, p = 0.04, respectively). Impaired endothelial function was also associated with increasing BH_2_ in all malaria patients (r = -0.42, p = 0.03) and those with severe malaria (r = -0.48, p = 0.04) but not in the MSM group alone. The association between endothelial function and BH_2_ remained significant after controlling for disease severity (partial correlation coefficient = -0.37, p = 0.04). Evaluation of markers of endothelial activation showed that ICAM-1 levels were positively associated with BH_2_ (r = 0.4, p = 0.02) and inversely associated with BH_4_ (r = -0.38, p = 0.04) and the BH_4_/BH_2_ ratio (r = -0.52, p = 0.003) in all malaria patients, but only with BH_4_/BH_2_ (r = -0.63, p = 0.03) in the SM group. The association between ICAM-1 with BH_4_ (partial correlation coefficient = -0.38, p = 0.035) and the BH_4_/BH_2_ ratio (partial correlation coefficient = -0.40, p = 0.03) remained significant after adjustment for malaria severity. The level of angiopoietin-2, another marker of malaria severity, was associated with increasing BH_2_ (r = 0.44, p = 0.02), but was not significant after adjusting for disease severity.

### Plasma phenylalanine, and BH_4_, BH_2_, and the BH_4_/BH_2_ ratio

Plasma phenylalanine levels were significantly increased in SM (median 176 μmol/L, IQR 85–250) compared to MSM (101μmol/L; IQR 84–110), SS (114μmol/L; IQR 112–332), and HCs (54μmol/L; IQR 51–58); Kruskal-Wallis p<0.001 (Table 2). Among all patients with malaria, plasma phenylalanine levels were inversely related to the BH_4_/BH_2_ ratio (r = -0.44, p = 0.04, including after controlling for disease severity [partial correlation coefficient = -0.38, p = 0.04]) and positively related to BH_2_ levels (r = 0.39, p = 0.03, including after controlling for disease severity [partial correlation coefficient = 0.48, p = 0.02]), but not BH_4_, B_0_, NH_2,_ or N_0_.

## Discussion

In adults with falciparum malaria, urinary tetrahydrobiopterin (BH_4_) was decreased and 7, 8-dihydrobiopterin (BH_2_) increased in proportion to disease severity, and a decreased BH_4_/BH_2_ ratio was associated with an increased risk of severe disease. The BH_4_/BH_2_ ratio is a reliable correlate for endothelial cell-dependent NO synthesis in vascular diseases [14–16]. The finding of an association between decreased BH_4_/BH_2_ ratio and increased BH_2_ with impaired microvascular reactivity and increased endothelial activation is consistent with a mechanistic role for oxidative stress and vascular NOS dysfunction. The association of increased BH_2_ and low BH_4_/BH_2_ ratios with increased phenylalanine levels suggests that systemic deficiency of BH_4_ causes impaired phenylalanine hydroxylase function as well as NOS dysfunction in malaria.

We have previously shown decreased systemic NO production in both African children and Indonesian adults, proportional to disease severity [5,6]. In adult falciparum malaria, there is also decreased endothelial and pulmonary NO bioavailability associated with low levels of the NOS substrate L-arginine [6], increased levels of the endogenous NOS inhibitor asymmetric dimethylarginine (ADMA) [10], NO quenching by cell-free hemoglobin [11] and L-arginine reversible endothelial dysfunction in moderately severe malaria [6]. The role of the key NOS cofactor, BH_4_, has not hitherto been shown in human malaria. In a recent murine severe malaria model, uncoupling of NOS with increased production of superoxide and impaired microvascular perfusion has been observed, and this was partially reversed by administration of intravenous BH_4_ [19]. Our results suggest that uncoupling of NOS due to decreased BH_4_ bioavailability and increased BH_2_, is also a key mechanism of impaired NO bioavailability in human severe falciparum malaria and in pathogenesis of severe disease.

The physiological role of NOS is oxidation of L-arginine and oxygen reduction to produce NO and citrulline [14,15,20]. BH_4_ regulates the coupling of the heme-oxygen intermediate to oxidation of L-arginine in NOS, and deficiency of BH_4_ as a co-factor can result in the output changing from NO to superoxide [14,15,20]. Increased oxidative stress can convert BH_4_ to the oxidized form BH_2_, with the decrease in BH_4_ increasing superoxide, resulting in a feed forward cycle with further oxidization of BH_4_ to BH_2_ [16]. Since BH_2_ can serve as a competitive inhibitor at the BH_4_ binding site in NOS, the BH_4_/BH_2_ ratio is likely to determine NOS coupling in malaria and determine the relative proportions of NO and superoxide production, as others have observed *in vitro* [16].

Systemic bioavailability of BH_4_ depends on three pathways of pterin metabolism. First is *de novo* synthesis from GTP. A second is regeneration of BH_4_ from quinonoid dihydrobiopterin by dihydropteridine reductase (e.g in hepatocytes for phenylalanine hydroxylase activity) and third is the salvage of 7,8 dihydrobiopterin (BH_2_) back to BH_4_ via dihydrofolate reductase (important for NOS activity in endothelial cells). We found no diminution of total biopterins excreted, suggesting that mechanisms controlling overall biopterin production are not impaired. Instead the decrease in BH_4_ associated with severe malaria appeared to result from its oxidation coupled with inadequate reduction of BH_2_ to BH_4_. *In vivo* recycling of BH_2_ to BH_4_ is the main regulator of the BH_4_:BH_2_ ratio, which in turn controls NOS coupling [16].

Our urine collection procedure allowed for capture of pterins, both biopterins and neopterins, in their excreted oxidation states. Our liquid chromatography methods allowed quantification of both dihydroneopterin and neopterin, the reduced and oxidized metabolites found in humans. This was of interest because these measurements provided information, in addition to biopterins redox status, on the partitioning of oxidized and reduced neopterins. We expected high total neopterin values in malaria and in septic patients and indeed this was found (Table 2). Elevated total neopterin has been reported previously and is the result of interferon-gamma-induced macrophage/monocyte activation with transcriptional induction of *GCH1* mRNA [21]. Mononuclear phagocytes have extremely low pyruvoyl tetrahydropterin synthase (PTPS) activity. Consequently the product of GTPCH catalysis, 7,8 dihydroneopterin triphosphate, accumulates, is dephosphorylated intracellularly, and diffuses to extracellular fluid and then plasma as NH_2_. Neopterin in healthy controls is excreted primarily as reduced dihydroneopterin (NH_2_:N_0_ = 4.4). In patients with severe malaria, despite marked elevation in urinary levels of total neopterins, the portion excreted as NH_2_ fell significantly (NH_2_:N_0_ = 2.0). Importantly the oxidation of NH_2_ to N_0_ is non-enzymatic. This suggests a milieu of oxidative stress in SM. It provides additional support for the redox imbalance observed for the biopterins, that is a fall in the ratio of reduced to oxidized metabolites.

An increase in oxidative stress has been observed in Bangladeshi adults with severe falciparum malaria [22]. This may explain the increased conversion of BH_4_ to BH_2_ as seen in this study, with the decreased BH_4_/BH_2_ ratio suggesting impaired recycling of BH_2_ to BH_4_ in severe malaria. Similar to certain cardiovascular diseases [16], our results suggest it is the BH_4_/BH_2_ ratio and not BH_4_ or BH_2_ alone that reflects NOS coupling in malaria. A decreased BH_4_/BH_2_ ratio was associated with an increased risk of severe disease, while decreased BH_4_ or BH_2_ alone were not associated with risk of severe disease. The association of a decreased BH_4_/BH_2_ ratio with impaired microvascular reactivity and endothelial activation, both previously shown to be associated with increased mortality in malaria, suggests that NOS coupling has an important role in determining malaria severity.

Our results also show that there is impaired microvascular reactivity and increased endothelial activation in severe sepsis, as we have shown previously [23,24]. However, it is notable that sepsis patients had *high* BH_4_ levels and *high* BH_4_/BH_2_ ratios compared to control subjects and malaria patients. The findings of increased BH_4_ levels in sepsis are similar to results from a previous study in which plasma biopterin levels were measured with high performance liquid chromatography [25]. The mechanism(s) of impaired vascular function in these sepsis patients is unclear, but does not appear to be related to impaired BH_4_ bioavailability. Furthermore, the high BH_4_/BH_2_ ratio in sepsis indicates that the low BH_4_/BH_2_ ratio in severe falciparum malaria is not simply a result of a nonspecific pathogen-wide systemic inflammatory response.

Results from our study also suggest that, in addition to low plasma L-arginine concentrations, increased ADMA and impaired NOS2 expression in severe malaria [5,6,8,10], decreased BH_4_ and increased BH_2_ can also affect NO bioavailability by altering NOS function in malaria. While increased L-arginine clearance in SM was seen in our pilot study of low dose L-arginine in severe malaria [12], decreased BH_4_ and increased BH_2_ could result in low NO despite the presence of normal levels of L-arginine. Results of studies with higher doses of L-arginine in severe falciparum malaria (ACTRN 12612000571875) are awaited, but future studies in severe malaria targeting hypoargininemia may need to consider simultaneously increasing both L-arginine and BH_4_ to increase NO production by NOS. Use of intravenous BH_4_ in patients with endothelial dysfunction associated with hypercholesterolemia and smoking results in acute improvement in endothelial NO production [26,27]. However, a randomized controlled trial of oral BH_4_ in patients with coronary artery diseases found that BH_4_ administration only resulted in increased conversion of BH_4_ to BH_2_ with no beneficial effects in clinical outcome [28]. Using anti-oxidants as adjunctive agents in severe malaria could also increase the BH_4_/BH_2_ ratio, but a recent trial using intravenous N-acetylcysteine (without BH_4_) in adult severe malaria did not show a benefit in clinical outcomes [22].

BH_4_ also plays a role as a co-factor for the enzyme phenylalanine hydroxylase, which converts phenylalanine to tyrosine [17]. As previously shown [17], both adults and children with clinical malaria are almost invariably hyperphenylalaninemic at presentation, which originally suggested a deficiency of BH_4_ in these patients. Blood levels of phenylalanine are normally tightly regulated between 30–80 μM by the BH_4_-dependent phenylalanine hydroxylase (PAH) in the liver [17]. The skewed BH_4_/BH_2_ ratio and high BH_2_ levels in these subjects correlated significantly with hyperphenylalaninemia. Hyperphenylalaninemia in SM is a transient acute abnormality, and it is relatively mild compared to the high levels observed chronically in untreated infants with phenylketonuria, a condition leading to severe brain damage caused by the direct toxicity of phenylalanine [29]. While it is not clear if the resulting hyperphenylalaninemia in malaria (especially cerebral malaria) is clinically relevant, it provides important supportive evidence for the functional significance of impaired BH_4_ bioavailability on BH_4_-dependent enzyme function in severe malaria.

This study has several limitations. The relatively small number of patients in each group and the small number of deaths in the SM group do not allow us to examine the independent effect of the biopterin metabolites on mortality or adjust for confounding variables. The numbers were however sufficient to demonstrate significant differences between groups. Also, the use of urinary measures of pterin metabolites as a measure of systemic biopterin bioavailability may not fully reflect intracellular concentrations in specific organs, though urinary biopterin quantitation has been shown to reflect systemic biopterin bioavailability [30–33]. It is possible that urinary BH_2_ and BH_4_ quantitation may be affected by renal function, although there was no association between blood creatinine and urinary BH_4_, BH_2_, N_0_ and NH_2_ in patients with malaria. Furthermore, measurement of the urine BH_4_/BH_2_ ratio is independent of creatinine excretion and is therefore not confounded by renal impairment. Importantly, the specialized collection techniques and assays we have used to measure urinary biopterin metabolite levels allow us to exclude artefactual *ex-vivo* oxidation.

In summary, the BH_4_/BH_2_ ratio is decreased in severe falciparum malaria but not in severe sepsis, and it is associated with an increased risk of severe disease, impaired microvascular function and endothelial activation, probably secondary to NOS uncoupling. The elevated levels of BH_2_ suggest that increased conversion of BH_4_ to BH_2_ due to increased oxidative stress and insufficient recycling of BH_2_ back to BH_4_ are the mechanisms of the low BH_4_/BH_2_ ratio in severe malaria. Our findings identify an additional mechanism of impaired NO bioavailability in severe falciparum malaria and pose an additional challenge to NOS-based adjunctive interventions to increase NO bioavailability in severe malaria.

## Methods

The study was undertaken at the Mitra Masyarakat Hospital, Timika, Papua, Indonesia, an area with unstable malaria transmission [34]. Patients ≥18 years of age with severe (SM) or moderately severe (MSM) *Plasmodium falciparum* malaria, or severe sepsis (SS) were enrolled as previously described [18]. SM was defined as peripheral parasitemia with ≥1 modified WHO criterion of severity [35], and MSM was falciparum malaria with fever within the past 48 hours, parasite counts of >1000/μL, requiring admission because of inability to tolerate oral therapy, but without WHO warning signs or severe criteria as previously described. Healthy controls (HC) were non-related hospital visitors without fever in the last 48 hours and no parasitemia. As an additional control for SM, patients with severe sepsis (SS) were also enrolled, defined as clinical evidence of infection, three or more features of the systemic inflammatory response syndrome, and evidence of one or more organ dysfunction, with or without septic shock, according to American College of Chest Physicians criteria, with no parasites by microscopy and a negative rapid diagnostic test for malaria [18]. All patients were managed by non-study hospital physicians and treated accordingly with antimalarials and antibiotics using hospital protocols.

A standardized history and physical examination were documented. Venous blood was collected on enrolment to measure biomarkers of severity, including lactate and plasma histidine rich protein 2 (HRP2), a measure of parasite biomass [36,37]. Plasma was obtained within 20 minutes and stored at -70°C for later quantitiation of the NO-dependent measures of endothelial activation, ICAM-1 and angiopoietin-2 by ELISA, as previously described [37]. Parasite counts were determined by thick and thin film microscopy. Hemoglobin, biochemistry, acid-base parameters, and lactate were measured with a bedside analyzer (i-STAT Corp). Reactive hyperemia peripheral artery tonometry (RH-PAT) was used to measure endothelial NO bioavailability as previously described [6,18]. RH-PAT uses finger probes to measure digital volume changes measured by a pressure transducer before and after application of an ischemic stress using a vascular cuff inflated to 200mmHg for 5 minutes followed by rapid cuff release. The RH-PAT index is a measure of the volume change and is at least 50% dependent on endothelial NO production. Near infrared resonance spectroscopy (NIRS) measurements were performed concurrently to assess microvascular reactivity on enrollment as reported before [18]. In brief, a clinical spectroscope (InSpectra 650, Hutchinson Technology) was used noninvasively to assess microvascular reactivity by measuring differential absorption of oxy (O_2_Hb) and deoxyhaemoglobin (HHb), which is then displayed as tissue oxygen saturation (ratio of O_2_Hb/O_2_Hb+HHb signals). By inducing an ischemic stress as detailed above, microvascular reactivity is the rate of skeletal muscle reoxygenation, defined as the rate of increase in StO_2_ in the first 14 seconds after release of occlusion. According to Beer’s law, this is confined to arterioles, capillaries, and venules of skeletal muscle with minimal interference from skin blood flow and myoglobin.

### Measurement of urine pterin compounds

Measurement of urine pterin concentrations, expressed as biopterins and neopterins in micromoles per millimole urine creatinine are used for diagnosis of gene mutations leading to BH_4_ synthesis, recycling and salvage deficiencies and reflect systemic pterin bioavailability [30–33]. BH_4_ is unstable and spontaneously oxidizes to its inactive metabolites, dihydrobiopterin (BH_2_) and to a lesser extent fully oxidized biopterin (B_0_) [38,39]. To prevent ex vivo spontaneous oxidation, urine was collected, via voluntary micturition or immediately after insertion of a Foley catheter, directly into vials containing the antioxidant pterin stabilizers 1,4-dithioerythritol (DTE) and diethylenetriaminepentaacetic acid (DETAPAC) [38,39] (as described in S1). Urine was then frozen at -70°C, and shipped in liquid nitrogen to Medical Neurogenetics Laboratories, LLC, Atlanta, GA United States. Concentrations of biopterin, 7,8-dihydrobiopterin, 5,6,7,8-tetrahydrobiopterin, and neopterin were quantified by high performance liquid chromatography using sequential electrochemical and fluorescence detection, as previously described [38,39]. Concentrations of pterin metabolites were normalized to creatinine concentrations in millimoles.

### Statistical methods

Statistical analysis was performed using STATA 11 software. The sample size for the patients with severe malaria was calculated from our previous study comparing RH-PAT in adults with severe and uncomplicated malaria with controls [6]. Using the difference and standard deviations found in RH-PAT index between severe malaria and controls, we estimated that a sample size of 14 in each group would have 80% power to detect a 25% difference between these two groups. Intergroup differences among malaria (MSM and SM) and controls were compared by ANOVA or Kruskal-Wallis test, where appropriate, with Wilcoxon Rank-Sum test used for pairwise comparisons. Pearson’s or Spearman’s correlation coefficients were determined depending on normality of distributions. Partial correlation coefficients were calculated adjusting for malaria disease severity. Logistic regression was used to determine the association between binary outcomes and goodness-of-fit was assessed by the Hosmer-Lemeshow test. A two-sided value of p<0.05 was considered significant.

### Ethics statement

The study was approved by ethics committees of the National Institute of Health Research and Development, Indonesia, and the Menzies School of Health Research, Australia. Written informed consent was obtained from patients or relatives if patients were comatose or too ill to give informed consent. Specific approval for this was obtained from both ethics committees.

## Supporting Information

S1 Study Protocol The protocol used in the conduct of the study. (DOC)
